# Development of Bio-Based Materials: Synthesis, Characterization and Applications

**DOI:** 10.3390/polym14173599

**Published:** 2022-08-31

**Authors:** Clara Delgado-Sánchez, Adrián Tenorio-Alfonso, Esperanza Cortés-Triviño, Antonio M. Borrero-López, Concepción Valencia

**Affiliations:** Pro2TecS-Chemical Product and Process Technology Research Centre, Universidad de Huelva, 21071 Huelva, Spain

The need to find suitable biomaterials and procedures from alternative products able to imitate or even enhance the performance of currently used products has become an important focus of research today due to the depletion of non-renewable resources and the increasing concern related to climate change, sustainability and environmental preservation. Thus, this book gathers different original articles and review manuscripts concerning the Special Issue **“Development of Bio-Based Materials: Synthesis, Characterization and Applications”**. The development of partial or fully bio-based materials has been included, with excellent outcomes in many different applications, as well as alternative procedures that can reduce the carbon footprint or optimize both production and energy consumption.

Given the interest in these materials, this book, including 27 articles and reviews written by research experts in their topics of interest, reports the most recent research on bio-based materials, with emphasis on the pharmaceutical and medical fields but covering a very extensive range of applications. Several novel and fascinating methods and studies related to thermo-responsive photopolymers (Figure 1A), biopolymer encapsulations, biopolymer-based films (Figure 1B), natural fibres production, biocompatible adhesives (Figure 1C), green composites (Figure 1D) and many other different materials have been introduced. The aim of this Special Issue, and now this book, is to provide a clear picture of the latest frontiers reached in the biomaterials field and their latest applications such as 3D bioprinting inks, immobilization of enzymes (Figure 1E), lubricating greases, elastomers and adhesives (Figure 1F), in which bio-based materials show great potential.

Emphasis on the biomaterial itself, the protocol followed, characterization and/or application have been reported, therefore contributing to a greener picture through the formulation of different more environmentally friendly products, but also to an understanding of the composition and structure of those systems, as well as applications thereof.

## Figures and Tables

**Figure 1:**
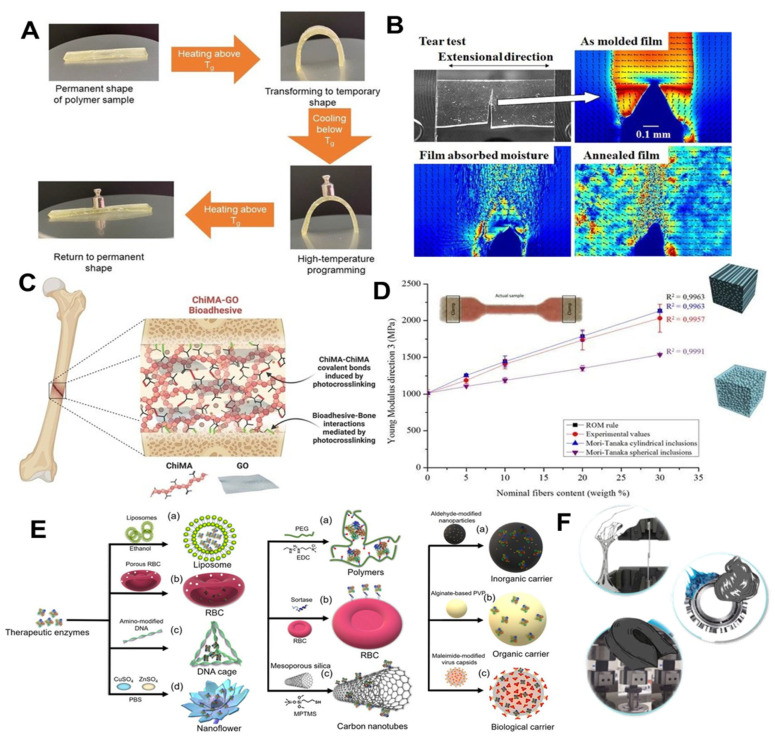
(**A**) Scheme of shape-memory characteristics of the bio-based polymers developed by Jaras et al. [1]. (**B**) Images of the in situ retardation measurements at the tip of the tear for the polylactic acid-based film developed in Yutaka et al. [2]. (**C**) Schematic overview of the bioadhesive formulation and application in Céspedes-Valenzuela et al. [3]. (**D**) Overview of the tensile properties of the green composites developed by Lemaire et al. [4]. (**E**) Different therapeutic enzymes studied in Zhu et al. [5]. (**F**) Adhesives, lubricating greases and elastomers as biomaterials studied in Borrero-López et al. [6].
